# Evaluation of the Zimbabwe HIV case surveillance pilot project, 2019

**DOI:** 10.11604/pamj.2020.37.353.25600

**Published:** 2020-12-17

**Authors:** Peter Nsubuga, Simbarashe Mabaya, Tsitsi Apollo, Ngwarai Sithole, Brian Komtenza, Takura Matare, Anesu Chimwaza, Kudakwashe Takarinda, Brian Moyo, Leon Mbano, Regis Choto, Thandekile Moyo, David Lowrance, Daniel Low-Beer, Owen Mugurungi, Alex Gasasira

**Affiliations:** 1 Global Public Health Solutions Limited Liability Company, Lilburn, United States,; 2 World Health Organization, Harare, Zimbabwe,; 3 Ministry of Health and Child Care, Harare, Zimbabwe,; 4 World Health Organization,Geneva, Switzerland

**Keywords:** HIV, case surveillance, informatics, electronic health records, continuum of care, patient monitoring

## Abstract

Zimbabwe has a high burden of HIV (i.e., estimated 1.3 million HIV-infected and 13.8% HIV incidence in 2017). In 2017, the country developed and implemented a pilot of HIV case surveillance (CS) based on the 2017 World Health Organisation (WHO) person-centred HIV patient monitoring (PM) and case surveillance guidelines. At the end of the pilot phase an evaluation was conducted to inform further steps. The pilot was conducted in two districts (i.e., Umzingwane in Matabeleland South Province and Mutare in Manicaland Province) from August 2017 to December 2018. A mixed-methods cross-sectional study of stakeholders and health facility staff was used to assess the design and operations, performance, usefulness, sustainability, and scalability of the CS system. A total of 13 stakeholders responded to an online questionnaire, while 33 health facility respondents were interviewed in 11 health facilities in the two districts. The HIV CS system was adequately designed for Zimbabwe’s context, integrated within existing health information systems at the facility level. However, the training was minimal, and an opportunity to train the data entry clerks in data analysis was missed. The system performed well in terms of surveillance and informatics attributes. However, viral load test results return was a significant problem. The HIV CS system was found useful at the health facility level and should be rolled out in a phased manner, beginning in Manicaland and Matabeleland South provinces. An electronic link needs to be made between the health facilities and the laboratory to reduce viral load test results delays.

## Project evaluation

### Introduction

The World Health Organisation (WHO) released the first HIV patient monitoring (PM) guidelines in 2006, providing technical recommendations on the collection and use of individual-level data for patient care and tracking [1]. In 2015, the consolidated HIV strategic information guidelines recommended priority aggregate indicators for use in programme management and monitoring [2]. Most recently, in 2017, the PM guidelines were updated and expanded to address HIV case surveillance (CS) and unique identification (UID) in the context of person-centred patient monitoring [3]. The latter included 15 priority recommendations for implementation of PM, CS, and UID while emphasising the fundamental inter-relationship between individual-level and aggregate data and data systems. When fully implemented, HIV case surveillance is fundamentally linked to PM data. However it is differentiated from PM in three important ways: i) whereas PM systems typically begin data collection at time of enrolment in HIV care and treatment, case surveillance starts at the time of HIV diagnosis; ii) whereas PM data systems often include unique identification (either unique identifiers, patient identifying information, or combinations thereof) for use at the facility level, case surveillance entails program-wide matching and de-duplication of patient records, thereby providing more robust testing, linkage, treatment/retention, and viral load figures than can be obtained through aggregated data and iii) compared with PM data, which supports the need of care providers, CS data represent a minimum and priority dataset which facilitates data management, analysis and use in programme management.

By 2019, HIV CS had not been widely implemented in sub-Saharan Africa, where the burden of the disease has been highest. In fact, among low-income countries with large, generalised epidemics, only Haiti has implemented case surveillance at a national scale [4,5]. Yet, major donors such as the Global Fund and PEPFAR are investing in case surveillance. With an increasing number of countries reaching 90-90-90 goals and epidemic transition, CS represents a potentially cost-effective method for collection and use of more robust, granular health data to improve programme management, outcomes, and impact in sub-Saharan Africa [4,6]. Globally, Zimbabwe is among the countries with the highest HIV burden, with an estimated 1.3 million people living with HIV [7]. Recent survey data indicate substantial progress on coverage of identification, treatment, and viral load suppression among PLHIV, positioning the country to achieve the 90-90-90 UNAIDS targets by 2020 [8]. A pilot phase of HIV CS implementation was commissioned in two districts of the country (i.e., Umzingwane and Mutare) to run from April 2017 to December 2018, to guide implementation of CS and help inform the subsequent rollout. During the period August 2017 to May 2018 a combined total of 19,454 clients were tested for HIV in the two districts with a positive yield of 6% of which 80% were notified and of those notified 71% were initiated on antiretroviral therapy (ART) on the same day. At the end of the pilot phase, the Ministry of Health and Child Care (MOHCC) an evaluation of the HIV CS pilot was performed to inform further steps. This report summarises the evaluation methods, findings, conclusions, and recommendations.

### Methods

**Setting**: at the time of the evaluation, there were 1574 facilities offering HIV services in Zimbabwe; of these, 624 (40%) high volume sites have an electronic patient monitoring system (ePMS) while the remaining sites use paper-based tools. The ePMS system was rolled out since 2013, as a complement to the paper-based patient monitoring system that uses a client intake form for all patients and a facility held OI/ART Care Booklet (green book) for clients testing HIV positive. Data from paper forms are entered into the ePMS retrospectively. Data from ePMS sites is then pushed manually monthly to the national level for analysis and report sharing quarterly. MOHCC, through support from Global Fund, is in the process of establishing a macro (central) database in the national data centre which will connect all ePMS sites thereby providing inter-facility linkages and facilitate de-duplication of records. The unique identification code used (OI/ART number) is only available to HIV positive clients. The OI/ART number is viewed as unstable because it uses data points that change (e.g., facility and district numbers), and patient names may change or be misspelt. With the possibility of re-testers and self-transferring patients, it has been challenging to have an accurate record of the actual numbers of people living with HIV and accessing services in Zimbabwe. There is a plan to extract and upload deduplicated ePMS data to the DHIS2 database monthly. For sites without ePMS, data on monthly return forms are abstracted from the paper-based registers and the green-book and uploaded into DHIS 2 which produces district-level aggregate data. It is not possible to detect duplicate records in the aggregate DHIS2 data.

**Evaluation approach**: the evaluation was implemented in September 2019 and conducted by a team comprising Zimbabwe MOHCC and WHO staff; and led by an external consultant. We performed a cross-sectional study using mixed methods (quantitative and qualitative) and based on published public health surveillance evaluation methods [9,10]. We developed and utilised an evaluation design matrix which was used to create the evaluation questions. We used an online set of tools for stakeholders from the MOHCC national and sub-national levels and interviewer-administered questionnaires; for staff at participating health facilities in pilot districts. A single common form was completed for each health facility.

### Elements in the evaluation design matrix and evaluation questions

**Design and operations**: health information system components of the HIV CS model, including digital and paper tools, data flow and resource requirements.

**Performance**: system attributes, including simplicity, timeliness, acceptability, sensitivity, representativeness, data quality, stability, and informatics attributes, such as information quality, system quality, user experience and service quality.

**Usefulness**: use of CS data, achievements, implementation challenges and lessons learned.

**Sustainability and scalability**: views on the scalability and sustainability of the HIV CS system in Zimbabwe.

**Evaluation questions**: the following evaluation questions were used to guide the assessment: a) what is the design of the HIV CS pilot? b) what is the performance of the HIV CS pilot? c) has the HIV CS pilot been useful for HIV programming? d) if Yes to question 3, how can the MOHCC and its partners sustain and scale up the HIV CS?

**Figure 1:**
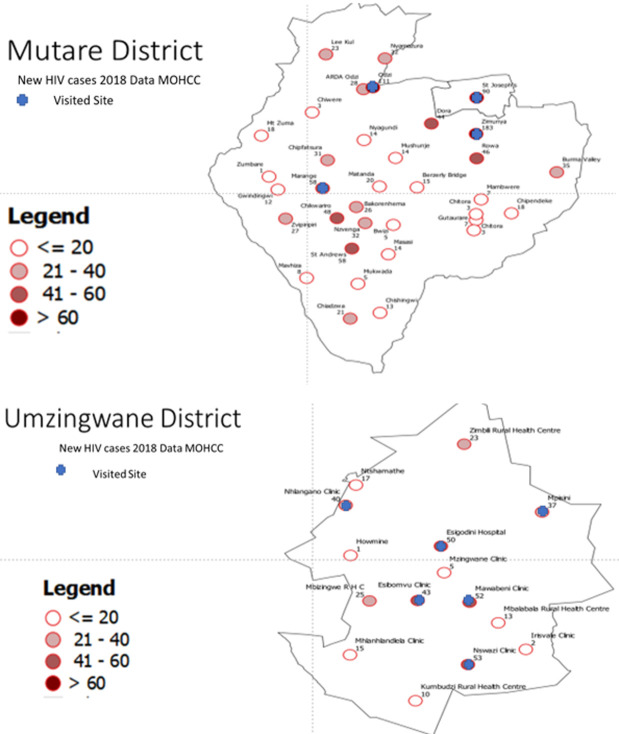
location of the health facilities that were visited in the 2019 HIV case surveillance evaluation in Mutare and Umzingwane Districts in Zimbabwe

The health facilities visited in the two pilot districts were selected based on the number of new HIV cases reported in 2018, starting with those that had the most substantial client volumes and could be visited within the evaluation period (Figure 1). At the health facilities, we interviewed the staff which included patient counsellor, the ART nurse, the data entry clerk (DEC) and the environmental health nurse as a group and the results are reported as group results. We observed how the health facilities conducted their activities. We analysed the data using Epi Info version 7 (US Centers for Disease Control and Prevention) to generate frequencies and proportions. An online word cloud generator word art [11] was used to summarise the qualitative responses. The evaluation was a public health evaluation and did not need ethical approval. The MOHCC provided written permission for the evaluation that was endorsed at the Provincial Medical Office before access to the districts and the health facilities.

### Results

A total of 13 (52%) stakeholders out 25 to whom the questionnaire was sent responded to the online survey. A total of 33 health facility respondents were interviewed at 11 health facilities, four from Mutare district and seven from Umzingwane (Figure 1).

**Design of HIV CS**: respondents reported that the HIV CS system was adequately designed for Zimbabwe´s context, leveraging existing system capacity and operations at the facility level, using the patient flow that was designed for ePMS. However, the training was minimal, only the health facility in-charges were trained for the most part, and an opportunity to train the data entry clerks to become data entry and analysis clerks was missed. District and provincial responsibilities were not clearly defined. The nurses and counsellors were also not trained adequately on the system; they relied on being trained by the facility in charge. The system also lacked geocoordinates, and a mapping module, to enable mapping of hotspot locations of new HIV diagnoses, high ART attrition, and viral load non-suppression. The respondents reported that the numerous forms and registers increased the burden of work on staff as the CS system added new forms on top of existing ePMS forms. Solar power backup was not available to all data entry sites; in some cases, it just needed a cable to be provided. Not all the data entry was linked to the MOHCC local area network.

**Figure 2:**
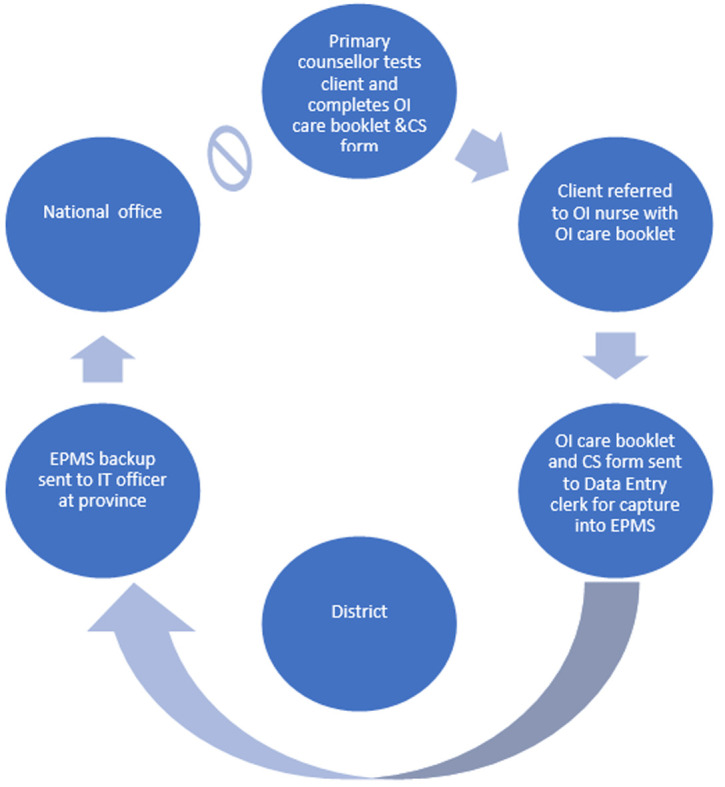
data flow in the HIV case surveillance system in Mutare and Umzingwane Districts, Zimbabwe 2019

**Data flow in the HIV CS system**: the HIV CS system had a paper-based and an electronic component (Figure 2). The paper-based component began with a client receiving HIV-testing services within a health facility. The primary care counsellor conducted testing, and the data are documented in several existing registers and forms [i.e., outpatient register (OPD), HIV testing service (HTS) register]. Once the client was determined to be HIV positive, another set of registers and forms were used (i.e., pre-ART, CS Form). The client was started on treatment by the nurse, at which time the opportunistic infections/antiretroviral treatment (OI/ART) booklet with the unique identifier (also called the OI/ART number) was given to the client. All the data were entered into the computer by the data entry clerk (DEC). The nurse and primary care counsellor managed follow-up appointments. The DEC entered the follow-up visit data. Data were sent monthly to the district using USB flash drives, and then by email to the province and then the national level IT team, which troubleshot issues using a WhatsApp group chat. There was no feedback in the system; summary reports from the national level were not sent back to the health facilities.

**Performance of the HIV CS**: the system (both paper-based and ePMS) performed relatively well in terms of the surveillance attributes and informatics attributes (Table 1, Table 2, Table 3). However, resources, for example, printing paper and printer toner cartridges were not available at all sites that we visited. Estimates of the underlying number of HIV positive clients in a catchment area were not available at the health facility level; therefore, they could not calculate where they were terms of the first 90 of the HIV cascades. Data analysis training had not been started at the health facilities for all the staff. Viral load results return was a significant problem with several viral load results delaying and negatively affecting patient management decisions.

**Table 1:** surveillance system attributes Zimbabwe HIV case surveillance pilot evaluation 2019 with measurements

Attribute/Definition	Measurement	Findings
**Simplicity:** the system´s structure and ease of operation	Health facility group response	9/11 (82%) reported the system is simple to use 5/11 (46%) reported the system adds to their workload DECs found the new ePMS challenging to use because of a lack of training
	Stakeholders views	9/13 (69%) believe the system is simple to use
	Observation	Health facility respondents could easily describe the client flow but reported it takes a lot of time (approximately 1 hour to enroll a new HIV positive patient) They also reported that the forms are too many
**Flexibility:** ability to adapt to changing information needs or technological operating conditions with little additional time, personnel or allocated funds	Health facility group response	Health facility respondents found the new CBS forms a bit difficult to use and missing some elements (e.g., syphilis tests) which meant a new register
	Stakeholders views	8/13 (62%) of stakeholders reported that the system is flexible
	Observation	Health workers found a way to use the new forms even without training
**Data quality:** the completeness and validity of the data recorded in the system	Health facility group response	All (100%) of health facilities visited entered their data into the computers within 2 days of a new HIV positive case
	Stakeholder´s views	12/13 (92%) of stakeholders believe that the system provides quality data that can be used for public health actions
	Observation	The data in the patient OI/ART booklet matched the data in the computers based on random checks Data moved up from health facilities, to districts, to provinces, and national level
**Acceptability:** the willingness of persons and organisations to participate in the system	Health facility group response	9/11 (82%) found the system acceptable to use
	Stakeholders views	8/13 (62%) believe the system is acceptable to users
	Observation	Health facility respondents generally reported that the system worked within their patient workflow However, several respondents thought CBS was a form, not a system
**Sensitivity:** the proportion of cases of a disease (or event) detected by the system. Is synonymous with completeness	Health facility group response	11/11 (100%) reported that anybody who wanted to test and take antiretroviral drugs in their community would come to their health facility since that is where all is antiretroviral treatment provided freely
	Stakeholders views	8/13 (62%) believed the system is sensitive enough to identify all HIV patients in the community and at health facilities
	Observation	The facilities provided all the HIV treatment in their areas One district had targets for testing that were found at the facilities. There was no target for the expected number of HIV positive (the first 90)
**Representativeness:** ability to accurately describe the occurrence of a health event over time, and its distribution in the population by place and person	Health facility group response	Health facilities could only describe the people who were tested at the facility and could not talk about the unknown number who never came for testing. 11/11 (100%) reported that anybody who wanted to test and take antiretroviral drugs in their community would come to their health facility
	Stakeholders views	Only 6/13 (46%) believed HIV PMS & CBS provides an adequate representation of all HIV infected patients that occur in the community and the health facilities. This is because the system is based on people showing up to be tested, or community testing which is variable
	Observation	There was no standardised form of time, place, person analysis Some DECs tried cascade analyses, but it was not based on any training
**Timeliness: t**he speed between steps in a system	Health facility group response	In all health facilities data were entered almost immediately after the clients are seen and sent monthly to the district for onward transmission
	Stakeholders views	9/13 (69%) believed HIV PMS & CBS provided timely notification of HIV patients at the health facility and in the community to the district
	Observation	District data were sent to the province and national level monthly Viral load testing had severe delays (in results coming back to the facilities) that may hamper treatment effectiveness There was no real feedback from the higher levels to the health facilities
**Stability:** the system´s reliability (ability to collect, manage, and provide data without failure) and availability (ability to be operational when needed)	Health facility group response	Only 1/11 health facility computers had crashed, meaning that 10/11 (91%) found the system stable
	Stakeholders views	Only 6/13 (46%) stakeholders believed HIV PMS & CBS is a stable system
	Observation	The computerized component of the system is stable since it is health facility-based However, Zimbabwe electric power grid outages in places where there is no solar (or solar connection) can be challenging

**Table 2:** surveillance system attributes Zimbabwe HIV case surveillance pilot evaluation 2019

Attribute	Summary Finding
**Simplicity**	The system was relatively simple although there is a lot of duplicated paperwork and forms
**Representativeness**	The system could test all those who want to be tested, but cannot easily find those who do not want to be tested
**Timeliness**	Intra-facility timeliness was very good, and the data flows up the health system on time. Viral load results are delayed
**Stability**	The paper-based system was stable, and the computerized system was stable where the is solar power backup, which not all facilities
**Acceptability**	The staff accepted the system, although there was a high workload
**Sensitivity**	The denominator of the HIV positives in the community was not clearly known, so sensitivity is not clear
**Data quality**	The system had high data quality both paper-based and computerized
**Flexibility**	The system was able to take on the new CBS form with almost no training, and the health workers made it work
**Predictive value positive**	Not assessed

**Table 3:** informatics attributes Zimbabwe HIV case surveillance pilot evaluation 2019

Attribute cluster	Summary Finding
**Information quality**	The system was accurate but not complete because it lacked geocoordinates The data were relevant and consistent
**System quality**	Users understood the system but needed training on the system updates The system mirrored the workflow in the clinic and was available as a standalone system through laptops. It could easily be modified if there was a change and was running on java which eases the modification Use of MySQL and Python enabled ease of programming and merging of data. The system was just using a fraction of the installed storage capacity of the server and the local laptops It urgently needed to be linked to the Laboratory Information Management System to shorten the duration that viral load test results take Power challenges existed where there is no solar link to the HIV clinic
**User experience and service quality**	Users have a WhatsApp group which enabled them to troubleshoot issues However, none of the DECs we met had been formally trained on the system. DECS need to become DEACs (with analysis), basic analysis training (e.g., time, place, person, HIV-cascades) was urgently needed The National level office was also understaffed and used interns for some of the critical functions

**The usefulness of the CS**: the system was used at the health facility level to track the HIV positive clients in their catchment area; all facilities that were visited were aware of what was happening to their clients (Table 4). The system was being used to conduct simple analyses of the clients, both new and old, and preparing simple cascades. At the health facility level, the system has enabled the staff to order the exact number of antiretroviral drugs that were needed for their clients and thus minimise wastage. There was limited evidence of the system´s usefulness at district and provincial level.

**Table 4:** usefulness of HIV case surveillance system direct quotes from stakeholders Zimbabwe HIV PMS and case surveillance evaluation 2019

Respondent Number	Response
1	It is a useful system if the country is going to reduce the HIV prevalence further
2	The system has led to better management of HIV positive clients and improved follow up as the programme is interested in documenting the outcomes as per the cascade for the continuum of care
3	The system helps the country in identifying where new cases are coming from and risk factors. The addition of recency testing helps to identify where new infections are coming from. This will help the country to design prevention strategies. Old infections analysis help to design case-finding strategies. Analysis of the treatment cascade (other sentinel events) will help the country to identify leakages along the continuum of care and design appropriate response.
4	The system assists in management, tracking and follow up of patients inclusive of retention among other patient sentinel surveillance
5	We can easily find out where more infections are happening
6	Longitudinal tracking of clients with unique identifier will reduce duplication of services and be able to account for each client
7	The system will improve case tracking, tracing, follow up and documentation
8	HIV CS system is useful for characterising all notified cases by person, time and place which facilitates targeted (i.e., hotspot mapping) and efficient implementation of interventions at the population level
9	The longitudinal tracking of HIV positive clients with their sentinel events until death helps programmers to make informed decisions using reliable gathered information.
10	Indeed, the system is useful. The system is used to target intervention for HIV epidemic response
11	The system acts as a client tracking tool and also assists in that the facility can refer to CS when searching for a client using the ePMS System faster than searching through piles of registers.
13	The system looks at the holistic approach in the management of HIV positive clients

**Figure 3:**
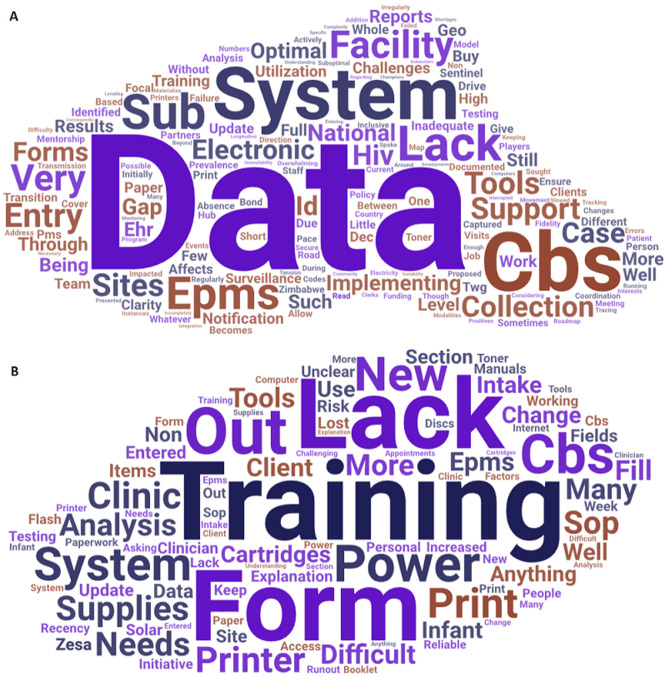
A) word cloud view of challenges from the stakeholder views Zimbabwe HIV CBS and PMS Pilot Evaluation 2019; B) word cloud view of challenges health facility views Zimbabwe HIV case surveillance pilot evaluation 2019

**Challenges with implementing the HIV PMS and CBS**: the significant challenges that were identified by the stakeholders in implementing the HIV CS system were the lack of data utilisation, training, and tools to support implementation (Figure 3A). Coordination among stakeholders was also identified as a challenge. At the health facility level, the main challenge was the lack of training on the system and a lack of supplies to operate the system (Figure 3B). Other problems included a lack of regular electricity. Respondents at 5/11 (45.5%) health facilities reported that there were too many forms and a lot of paperwork.

**Health facility views on scale-up**: respondents at all (11/11) health facilities believed that the system should be scaled up to the rest of the country. They reported that the country would lose the ability to track HIV clients and identify double testers if there was not a national linked system. They also said that a national system would also identify hotspots. They also reported that a national system would help in ordering the right number of antiretroviral drugs. The health workers believed that a national system would reduce the paperwork and forms.

**Stakeholders´ views on scale-up**: a total of 10/13 (77%) stakeholders thought that the system was an efficient option for Zimbabwe since it leverages existing digital platforms. A majority of 11/13 (85%) stakeholders thought the country would be able to roll out CS to all facilities with targeted partner support given the current funding challenges. The stakeholders believed that the country would lose the ability to conduct longitudinal tracking of HIV patients and individualised care if the system was not rolled out. The CS system allowed patient-level tracking of cases.

### Discussion

**Principal results**: this is the first report of findings from an evaluation of HIV case surveillance implementation in sub-Saharan Africa. In our evaluation of the HIV CS pilot in two districts in Zimbabwe, we found that the HIV CS system was adequately designed for Zimbabwe´s context, leveraging existing health information platforms and operations at the health facility level. We also found that the system (both paper-based and ePMS) performed well in terms of both surveillance informatics attributes, and that the data are used at the health facility level to ensure linkage from testing to treatment and track PLHIV on ART in their catchment area. All facilities that were visited were using these data to conduct essential cascade analyses, including both new and old clients. Almost all respondents reported that, with partner technical and financial support, the country can rollout the HIV CS system to all health facilities. Whereas the system was adequately designed, the training to staff was minimal, and an opportunity to train the DECs into DEACs (i.e., data analysis in addition to data entry) was missed. The nurses and counsellors were also not trained adequately on the system, and staff turnover led to a reduction in those who had primary training on the system. The system needs to include geocoordinates and related data analysis module should enable mapping of “hotspots” of new cases, ART retention and loss to follow up (LTFU), and viral load suppression/non-suppression based on granular health administrative units which facilitate programme response.

At the time of this evaluation, Zimbabwe was in the process of implementing electronic health records (EHR) system [12]; more effort needs to be made to reduce forms and registers as they increase the burden of work on staff; EHR, if implemented, would solve this. Solar power backup and or off-line applications needs to be available to all data entry sites, and more sites need to be linked to the MOHCC local area network, which can provide an easy way to upload health facility data but also provide feedback. The performance of the HIV CS could be improved if resources like paper and toner were made available to all sites; these resources would enable locally generated analyses to be printed and posted at the site for all to see progress or challenges. Also, if feasible estimates of the underlying number (i.e., the denominator) of HIV positive clients in a catchment area need to be provided to each facility from the district level to enable them to calculate where they are in terms of the first 90 of the 90-90-90 HIV cascades [13]. Data analysis training needs to be urgently started beginning with the existing pool of data entry clerks, and this is a low hanging fruit as these staff can also be used to analyse other health data (e.g., TB, malaria, maternal and child health, epidemic-prone diseases) that are generated at the health facility level; these analyses would lead to prompt data-based action at the local level. ART retention is an essential programmatic indicator, and case surveillance data should enable timely determination of LTFU and tracking to promote retention on ART. Viral load suppression is the single-most-important clinical outcome associated with both reduced mortality and reduced incidence [14]. In principle, one of the most important functions of a CS system is assuring that if test results indicate virologic non-suppression, they are followed up rapidly to ensure intensive adherence counselling, follow-up VL testing, and switch to alternative regimens, as appropriate. Late return of VL test results was a significant problem at all the facilities that we visited. Data transfer between the laboratory management information system and ePMS/CS needs to be strengthened, either by direct interoperability whereby VL results are sent electronically to ePMS, or indirect digital transmission, e.g. SMS.

The usefulness of the HIV CS was evident at the health facility level, where the system enabled them to order the exact number of antiretroviral drugs needed for their clients and thus minimise wastage. However, use in programme management at higher administrative levels - another important data use case-was not apparent. The small scale of the pilot likely limited utility at the provincial level, however, one would have anticipated data use at the district level, and this was not seen; it is possible that this was still viewed as a study, hence hampering system utility. We also believe that the relatively positive experience that Zimbabwe has had can inform and strengthen planned or similar efforts by other African countries given the low level of implementation CS in Africa to date [5]. We believe that the experience from this pilot can be used to scale-up first to all the facilities in Manicaland and Matabeleland South Provinces (i.e., where the pilot was conducted), then to the rest of the country. A countrywide HIV CS system is needed for effective tracking of, identification of new cases and hotspots, tracking of PLHIV identified and on ART, including for viral load suppression. However, substantial resources, staffing, and training will be required for scale-up. We found that the OI/ART number, even with its disadvantages, seemed to be the only feasible unique identifier because not everybody has or can have national identity cards (e.g., adolescents, non-Zimbabweans). If the planned EHR is implemented as planned, a new UID may be possible, perhaps incorporating elements of patient identifying information.

**Limitations**: interpretation and generalisation of the findings of this pilot are subject to some limitations. First, health facility respondents were also participants in the pilot and could have views that were influenced by their role in the program. There may also have been a natural bias to focus on program successes, although we tried to tease out other critical points to the questions, and we performed some observations. Secondly, although several attempts were made to obtain answers from all relevant stakeholders; some did not respond, and their answers could have been different from those who responded. Third, the evaluation was limited in time, and we could not look at every facet of the performance of HIV CS and PM system, and could have missed vital information. Finally, the evaluation questions required the respondents to have adequate recall of events that occurred in the past, and this could have had a bearing on the results. The team tried to triangulate several sources of information to limit the effect of recall bias. Finally, this was a primarily qualitative assessment with limited direct assessment of CS data.

### Conclusion

We recommended the following to the MOHCC based on the findings of our evaluation. First, the HIV CS system is useful at the health facility level and should be rolled out in a phased manner, beginning with all facilities in Manicaland and Matabeleland South. Lessons learned from the provincial rollout can be used for a nationwide scale-up. Secondly, training modules and courses should be developed for all the DECs to enable them to conduct data analysis (e.g., cascading, person, place time analyses) and data use to inform health facility-level interventions to monitor local response to care and treatment and HIV prevention. Consideration should be made to change the name from Data Entry Clerks to Data Entry and Analysis Clerks. Third, standard operating procedures, training, and regular support supervision and mentorship should be provided to the health facility teams in the operation of HIV CS. Fourth, regular feedback on the data collected should be provided from all levels back to the health facility, and resources (e.g., toner, paper, and other consumables) should be regularly provided. Solar power backup and link to MOHCC local area network should be strengthened for all health facilities where HIV CS is implemented.

We also recommended that an electronic link needs to be made between the laboratory information system (LIMS) and ePMS to facilitate the rapid turnaround of viral load test results to MOHCC local area network- linked health facilities, and ePMS should include geocoordinates and a mapping module. Finally, a workflow analysis should be conducted to reduce duplicative and redundant paperwork and registers at health facilities. Efforts should also be undertaken to utilise innovations in public health surveillance, including careful piloting and use of electronic systems given the electricity uncertainties in Zimbabwe, and other innovations as described by Groseclose *et al*. [15]. We believe that the HIV CS system as piloted in Zimbabwe if scaled up will provide a country-generated, locally adapted system that can assist Zimbabwe in its long fight against the scourge of HIV/AIDS.
